# Supplementary figures and images for: Data-driven analysis of biomedical literature suggests broad-spectrum benefits of culinary herbs and spices
Source: PLoS One. 2018 May 29;13(5):e0198030. doi: 10.1371/journal.pone.0198030 (PMC5973616; doi:10.1371/journal.pone.0198030)

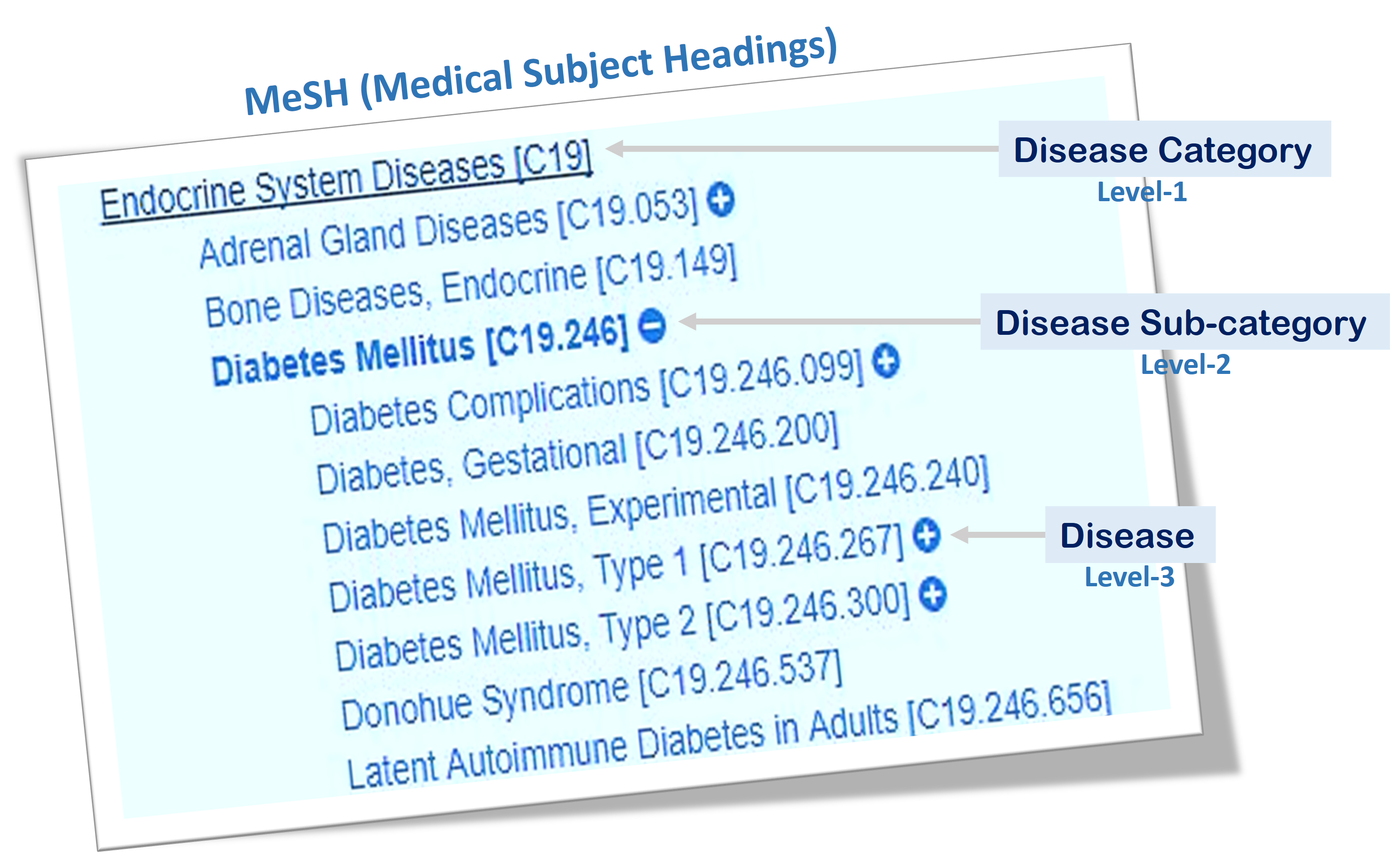

Supplement: S1 Fig — For the purpose of multi-level analysis, spices were associated with disease terms at three levels of MeSH hierarchy—‘category’, ‘sub-category’ and a ‘disease’. (TIF) [file pone.0198030.s001.tif]

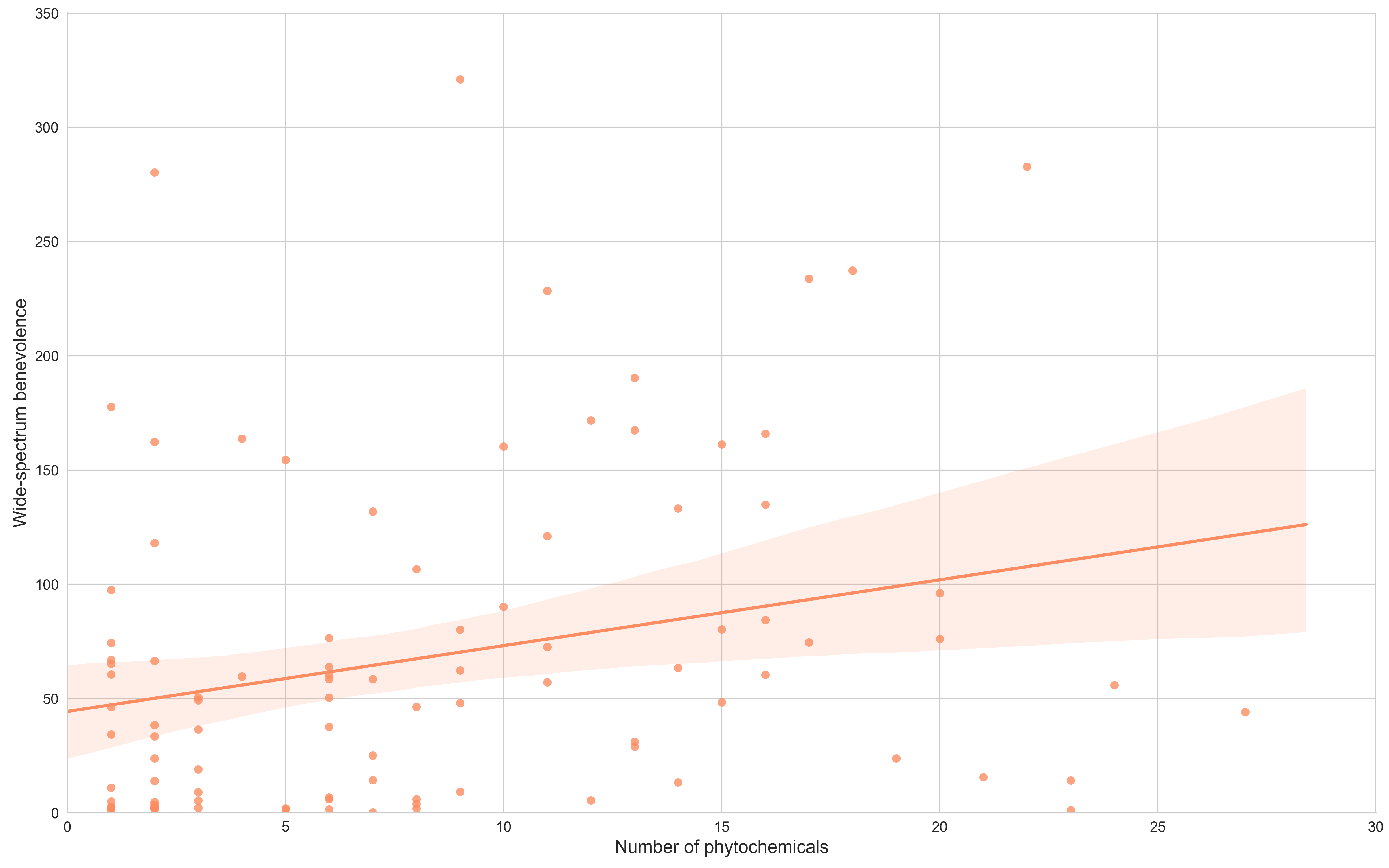

Supplement: S2 Fig — The data indicate that the broad-spectrum benevolence score of spices and their phytochemical repertoire are not correlated. (TIFF) [file pone.0198030.s002.tiff]

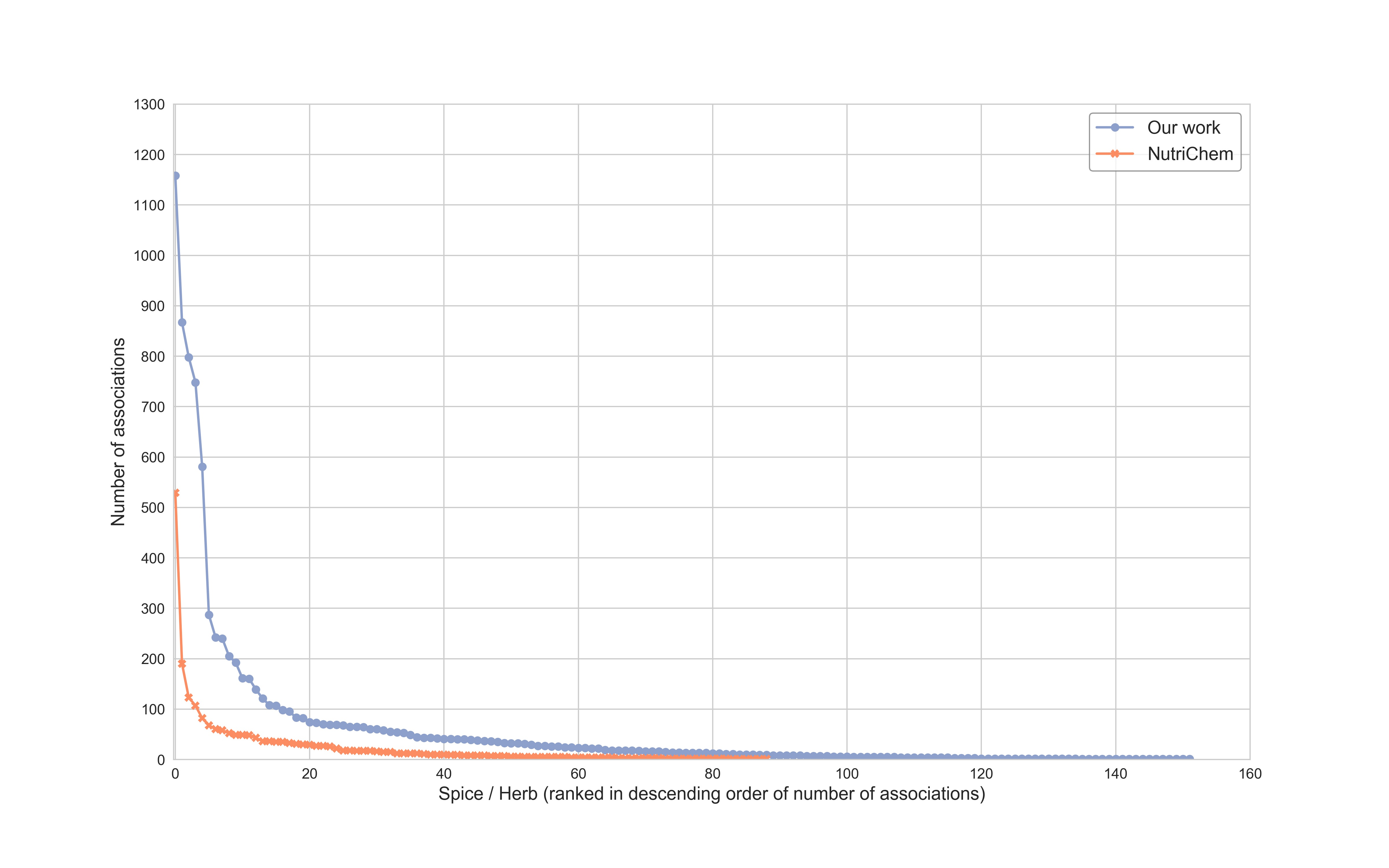

Supplement: S3 Fig — (TIFF) [file pone.0198030.s003.tiff]

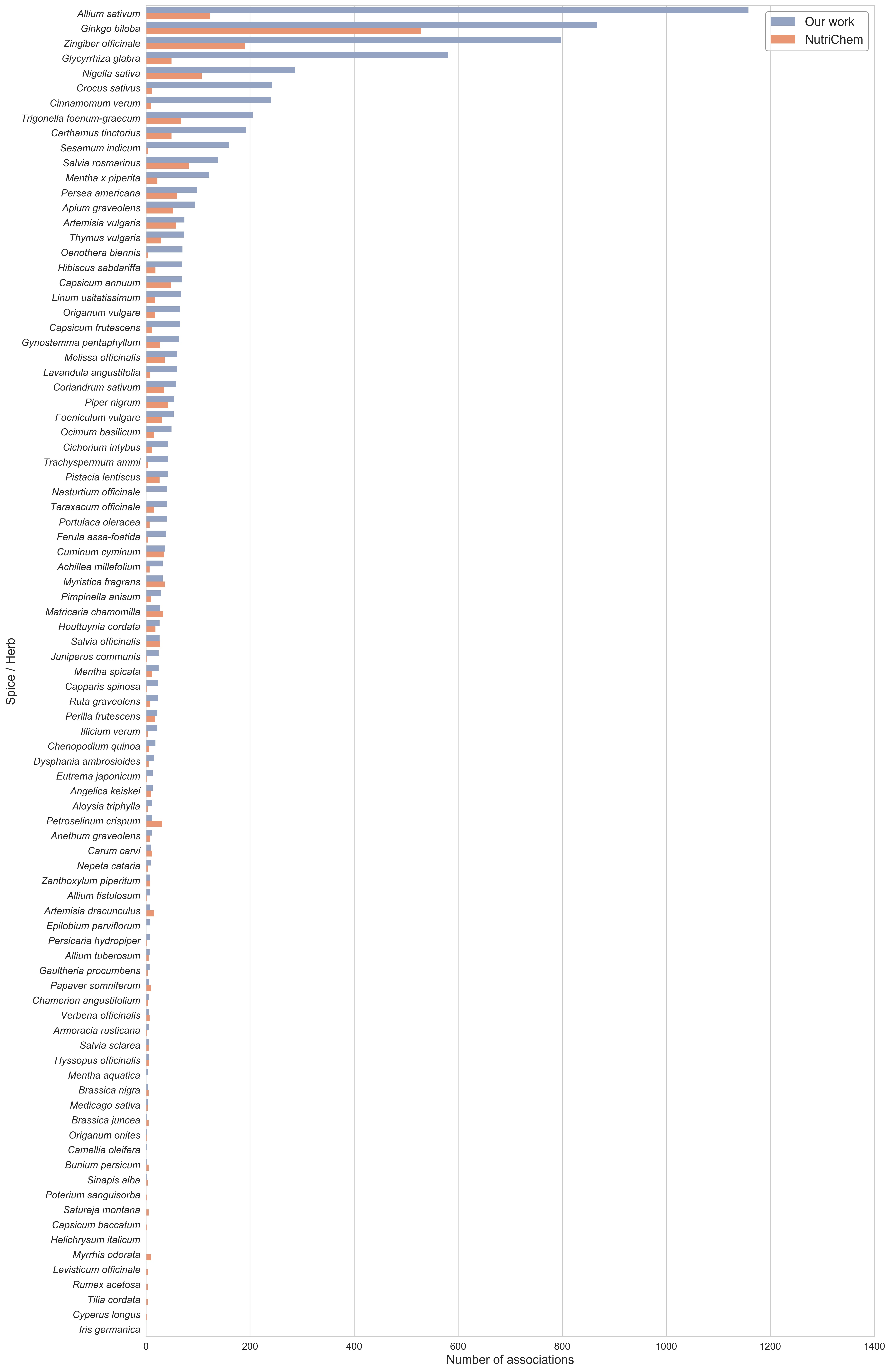

Supplement: S4 Fig — (TIFF) [file pone.0198030.s004.tiff]
