# Supplementary material for: Data-driven analysis of biomedical literature suggests broad-spectrum benefits of culinary herbs and spices
Source: PLoS One. 2018 May 29;13(5):e0198030. doi: 10.1371/journal.pone.0198030 (PMC5973616; doi:10.1371/journal.pone.0198030)
Supplement: S1 Table — (DOCX) [file pone.0198030.s005.docx]

**S2 Table:** Top ten broad spectrum spices and number of MeSH disease categories and subcategories with which they were positively associated.

| **Sr. No** | **Scientific Name** | **Positively Associated 'Disease Categories'** | **Positively Associated 'Disease Sub-categories'** | **Total number of Positive Associations** |
| --- | --- | --- | --- | --- |
| 1 | *Allium sativum* | 25 | 96 | 1092 |
| 2 | *Ocimum tenuiflorum* | 25 | 51 | 82 |
| 3 | *Curcuma longa* | 25 | 87 | 739 |
| 4 | *Zingiber officinale* | 24 | 81 | 795 |
| 5 | *Nigella sativa* | 24 | 66 | 278 |
| 6 | *Cinnamomum verum* | 23 | 55 | 220 |
| 7 | *Ginkgo biloba* | 23 | 82 | 830 |
| 8 | *Helianthus annuus* | 22 | 41 | 77 |
| 9 | *Carthamus tinctorius* | 22 | 49 | 182 |
| 10 | *Glycyrrhiza glabra* | 22 | 72 | 365 |
