# Supplementary material for: Data-driven analysis of biomedical literature suggests broad-spectrum benefits of culinary herbs and spices
Source: PLoS One. 2018 May 29;13(5):e0198030. doi: 10.1371/journal.pone.0198030 (PMC5973616; doi:10.1371/journal.pone.0198030)
Supplement: S2 Table — (DOCX) [file pone.0198030.s006.docx]

**S1 Table:** Hyper-parameters selected for the convolutional neural network Model 2 and Model 3.

| **Parameter** | **Model 2** | **Model 3** |
| --- | --- | --- |
| Filter sizes ($f_{z})$ | 2,3,4,5 | 3,4,5 |
| Number of filters ($n_{f}$) | 150 | 150 |
| Hidden units($n_{h}$) | 256 | 256 |
| Dropout probability ($p$) | 0.5 | 0.5 |
